# A method for efficient, rapid, and minimally invasive implantation of individual non-functional motes with penetrating subcellular-diameter carbon fiber electrodes into rat cortex

**DOI:** 10.3389/fnins.2026.1848862

**Published:** 2026-07-28

**Authors:** Joseph G. Letner, Jordan L. W. Lam, Miranda G. Copenhaver, Michael Barrow, Paras R. Patel, Julianna M. Richie, Jungho Lee, Hun-Seok Kim, Dawen Cai, James D. Weiland, Jamie Phillips, David Blaauw, Cynthia A. Chestek

**Affiliations:** 1 Department of Biomedical Engineering, University of Michigan, Ann Arbor, MI, United States; 2 Biointerfaces Institute, University of Michigan, Ann Arbor, MI, United States; 3 Department of Neurosurgery, University of Michigan, Ann Arbor, MI, United States; 4 Department of Electrical Engineering and Computer Science, University of Michigan, Ann Arbor, MI, United States; 5 Department of Cell and Developmental Biology, University of Michigan Medical School, Ann Arbor, MI, United States; 6 Neuroscience Graduate Program, University of Michigan, Ann Arbor, MI, United States; 7 Biophysics Program, University of Michigan, Ann Arbor, MI, United States; 8 Department of Ophthalmology and Visual Sciences, Kellogg Eye Center, University of Michigan, Ann Arbor, MI, United States; 9 Department of Electrical and Computer Engineering, University of Delaware, Newark, DE, United States; 10 Department of Robotics, University of Michigan, Ann Arbor, MI, United States

**Keywords:** carbon fiber, electrode fabrication, implantation, intracortical electrodes, neural probes

## Abstract

Distributed arrays of wireless neural interfacing chips with 1–2 channels each, known as “neural dust,” could enhance brain machine interfaces (BMIs) by removing wired connections through the scalp and increasing biocompatibility with their submillimeter size. Although several neural dust designs have emerged, currently reported procedures for implanting them in batches place the chips directly inside the brain, which can damage or displace large numbers of neurons. Therefore, a procedure for safely implanting neural dust in batches such that only ultrasmall microwire elements enter the brain is needed. Here, we demonstrate the feasibility of implanting batches of wireless motes that rest on the cortical surface and reach 1 mm brain depths via penetrating carbon fiber electrodes (6.8–8.4 μm diameter) without employing disruptive insertion shuttles. To simulate their implantation, we assembled over 230 mechanically-equivalent carbon fiber motes and affixed them to insertion tools with polyethylene glycol (PEG), a quickly dissolvable and biocompatible material. Then, we implanted batches into rat cortex *in vivo* and evaluated insertion success and their arrangement on the brain surface. When positioning motes for insertion, we discovered that they readily aggregated in molten PEG such that average array pitches were 5% longer than an individual mote’s dimensions (240 × 240 μm). Overall, 187/214 (87%) motes tightly-packed in 4 × 4 (*N* = 4) and 5 × 5 (*N* = 6) square grid configurations successfully inserted into rat cortex. After implantation, measurements of how much motes tilted (22 ± 9°, X̄ ± S) and had been displaced from their original positions were smaller than those measured in the literature for devices implanted inside the brain. Collectively, these data establish the mechanical viability of assembling and safely implanting motes with ultrasmall electrodes and epicortically-situated chips, motivating the use of arrays with similar geometries in future BMIs.

## 1 Introduction

Neural interfaces for recording and stimulating the nervous system have been used to study and treat a wide range of conditions (Ahnood et al., 2023). These include brain machine interfaces (BMIs), which can restore functions lost by neurological injury or disease (Losanno et al., 2023; Rubin et al., 2023), such as speech (Metzger et al., 2023; Willett et al., 2023) and walking (Lorach et al., 2023). Despite successful multi-year clinical trials (Rubin et al., 2023) and the recent regulatory success of newer systems (Drew, 2023), BMIs have yet to gain the approval required to become a broadly applicable clinical treatment (Patrick-Krueger et al., 2025). Challenges associated with, but not limited to, BMI hardware must first be mitigated (Lu et al., 2012), the most pressing of which is expanding the number of electrode channels in a safe and manageable way to interface with more neurons (Perna et al., 2023).

Large networks of neural interfacing chips that are both submillimetric and wireless could potentially solve these hardware issues (Ahmadi et al., 2019). Often called “neural dust” after a pioneering design (Seo et al., 2016; Losanno et al., 2023), these chips are comprised of a single electrode and an application specific integrated circuit (ASIC) responsible for wirelessly harvesting power and communicating while stimulating or digitizing recorded neural signals. The wireless link removes a wired connection that is currently transcranial and commonly transcutaneous. This could be advantageous for the BMI user by reducing the risk of infection (Bullard et al., 2020), reducing foreign body responses (FBRs) from micromotion (Khalifa et al., 2021b), and could offer the user more freedom of movement by obviating the physical tether to a computer system (Miziev et al., 2024). Additionally, restoring naturalistic hand control through BMIs may necessitate recording from more than 100,000 neurons (Lebedev et al., 2011). At such channel counts, wired connectors become increasingly difficult to manage (Rapeaux and Constandinou, 2021; Chen et al., 2023). Even with 100 channels, the connector is a frequent source of device failure (Barrese et al., 2013; Szostak et al., 2021). Using a wireless connection, individualized neural dust electrodes can be implanted modularly in batches to increase coverage or more efficiently target brain regions of interest (Khalifa et al., 2021b; Rapeaux and Constandinou, 2021). Also, if they are sufficiently small, they may cause less damage and be more biocompatible than current arrays (Seymour and Kipke, 2007; Khalifa and Etienne-Cummings, 2023). Collectively, these potential benefits have garnered interest in this architecture and a large increase in innovative approaches (Seo et al., 2016; Ahmadi et al., 2019; Khalifa et al., 2019; Cortese et al., 2020; Lee S. et al., 2020; Lee et al., 2021; Tawakol et al., 2024).

Current BMIs often target neurons deep in cortex to obtain high-resolution neural firing patterns (Moran, 2010; Losanno et al., 2023). However, implanting neural dust to these cortical depths in a way that improves upon the safety and performance of existing electrodes is difficult. Since placing neural dust onto the brain surface will primarily record field potentials rather than spikes from individual neurons (Hill et al., 2018), some groups have proposed implanting them inside the brain (Cortese et al., 2020; Sigurdsson et al., 2020; Khalifa et al., 2021a). However, these devices are comparable in size to traditional silicon probes (Lim et al., 2022; Lee J. et al., 2024), which are widely known to induce a negative FBR and damage neurons over time (Biran et al., 2005; Salatino et al., 2017; Szymanski et al., 2021). As a result, histology surrounding neural dust placed inside cortex has pointed to a similar FBR (Sigurdsson et al., 2020; Khalifa et al., 2021c). It would be better to place the bulk of the device onto the brain surface instead, i.e., epicortical placement (Khalifa et al., 2021c), and use a smaller electrode extending from the chip to penetrate the brain.

In order to safely implant neural dust with this geometry, the electrodes will need to be sufficiently small and biocompatible to avoid eliciting the FBR observed with traditional probes. Prior work using epicortical dust with microwire electrodes ( ≥ 50 μm diameter) (Lee et al., 2021; Lee A. H. et al., 2024; Yeon et al., 2021; Khalifa et al., 2023) were not ideal since microwires of this size can also elicit a negative FBR (Winslow and Tresco, 2010; Prasad et al., 2012). Instead, using penetrating electrodes that have low bending stiffness and subcellular size can minimize tissue damage (Seymour and Kipke, 2007; Stiller et al., 2018). Many ultrasmall probes have exploited these principles to markedly reduce the FBR (Thompson et al., 2020). These include the nanoelectronic thread (NET) electrodes (Luan et al., 2017; Zhao Z. et al., 2023), flexible polymer electrodes (Yasar et al., 2024), mesh electrodes (Yang et al., 2019; Zhao S. et al., 2023), and carbon fibers (Kozai et al., 2012; Guitchounts et al., 2013). However, this higher biocompatibility typically comes at the cost of increased difficulty when inserting them into the brain, requiring one or more insertional aids (Thielen and Meng, 2021). Implanting a network of thousands of individual electrodes further compounds this difficulty (Szostak et al., 2021) and highlights the need for a biocompatible electrode that can easily penetrate.

**FIGURE 1:**
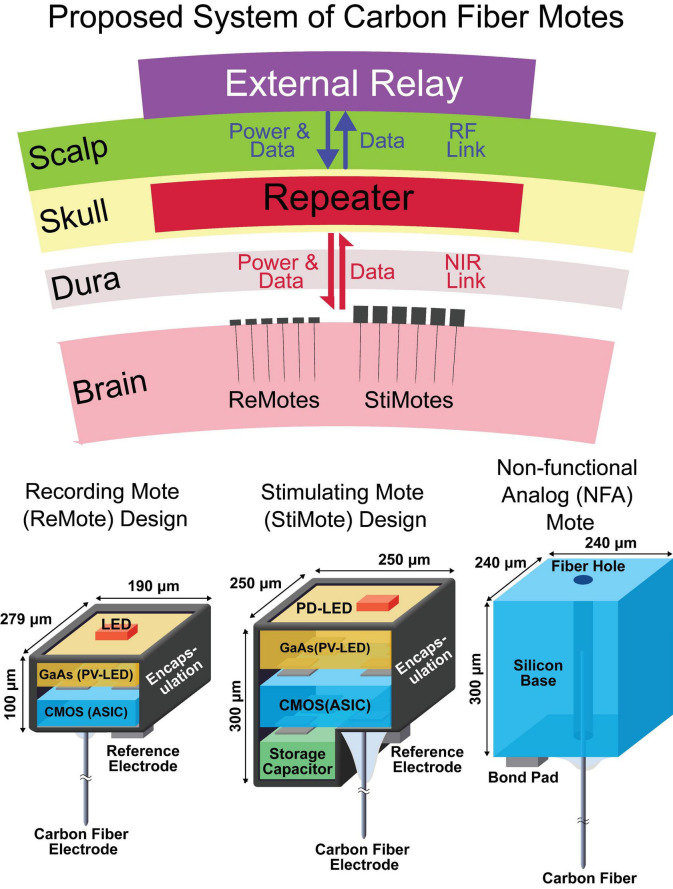
Proposed carbon fiber mote system. Top: Conceptual diagram showing a network of ReMotes and/or StiMotes recording or stimulating neurons, respectively. Chips sitting on the brain are powered by and communicate via a repeater unit embedded in the skull via NIR light. Carbon fibers penetrating the brain reach the targeted cells. The repeater is powered by and communicates via radiofrequency (RF) with an interface placed on the scalp. Bottom: Cross-sectional diagrams of the ReMote (left) and StiMote (middle) and the carbon fiber non-functional analogs used in this study to simulate those designs (right). For both functional designs, the top chip has a photovoltaic (PV) cell and light-emitting diode (LED) for wireless functionality, and an ASIC for device functionality below the PV-LED chip. Adapted from Lee J. et al. (2024).

Of the biocompatible electrodes listed above, carbon fiber electrodes (6.8 μm diameter) may be the easiest to insert, due mainly to carbon’s strength at small size (Patel et al., 2015). Our group has shown that chemically sharpened carbon fiber arrays reliably insert at least 1.5 mm into the brain without any insertional aids (Richie et al., 2024). We have also proposed (Lim et al., 2020) a neural interfacing system comprised of hundreds of wireless motes, each with its chip situated on the brain surface and a carbon fiber electrode penetrating to targeted cortical depths (Figure 1). These motes would be powered by and relay messages via near-infrared (NIR) light to a skull-mounted receiver (Moon et al., 2021; Costello et al., 2022), which would communicate with an external receiver placed on the scalp communicating with a computer (Figure 1). This proposed system has versions for recording neural signals (ReMote) and for stimulating neurons (StiMote) (Figure 1). Many of the system’s components have been validated separately (Lee J. et al., 2024), including both ASICs (Lim et al., 2022), (Lee J. et al., 2024), wireless uplink (Moon et al., 2021), (Atzeni et al., 2022), power harvesting (Sun et al., 2022), and carbon fiber electrode arrays (Patel et al., 2015, 2020; Richie et al., 2024). A critical requirement for clinically translating this system is a procedure for quickly implanting batches of motes with high throughput to avoid unnecessarily increasing neurosurgical complexity and duration (Szostak et al., 2021). However, previously reported approaches for implanting neural dust into the brain are not designed for delivering batches of epicortically-situated chips with ultrasmall penetrating electrodes (Khalifa et al., 2019, 2021a,^2023^; Cortese et al., 2020; Sigurdsson et al., 2020; Acebal et al., 2024; Lee A. H. et al., 2024). Therefore, here we designed a method for inserting carbon fiber mote batches and demonstrate the procedure’s surgical feasibility by using non-functional analogs with dimensions similar to the StiMote (Figure 1).

Here, we show the fabrication of carbon fiber non-functional analogs to determine viable carbon fiber assembly and implantation techniques for wireless designs. We produced more than 230 non-functional analogs consisting of a micromachined silicon base with an extruding carbon fiber and implanted them to test a novel method for swiftly, easily and efficiently implanting up to 25 carbon fiber motes into rat cortex *in vivo* simultaneously such that only the minimally-invasive carbon fibers penetrate cortex. Implanting motes using this method showed an 87% success rate (187 of 214 motes). Additionally, implanted motes had lower changes in spatial arrangement (22 ± 9° tilt angle, 65 ± 55 μm displacement) compared to intracortically placed designs as reported in the literature. To our knowledge, this work is the first to report methods for assembling epicortically-situated neural dust with ultrasmall penetrating shanks and is the first to introduce and validate a successful procedure for implanting such devices in batches, detailing the first steps toward their translation to clinical use.

## 2 Materials and methods

### 2.1 Fabrication of silicon bases for non-functional analog motes

Silicon bases were fabricated using standard cleanroom micromachining processes. These bases were designed to be prismatic with dimensions (240 × 240 × 300 μm, L × W × H) similar to proposed functional carbon fiber motes (Figure 1). The hole through the base, used for holding a protruding carbon fiber electrode, was designed to be conductive with connection to a backside metal pad for use as a wired mote. Ultimately, this functionality was not used. All steps are listed in Supplementary Note 1 to provide a full account of the motes’ material composition. Supplementary Figure 1 shows a cross-sectional diagram of the non-functional analog mote and comprising layers.

### 2.2 Affixing carbon fibers to mote bases

A process flow diagram of the assembly steps is shown in Supplementary Figure 2. Non-functional analog motes were assembled in batches that started with 15–20 motes. Bases were pulled off of the Parylene C layer holding them on the source wafer using forceps (Supplementary Figure 3A) and residual Parylene C was scraped off with a razor blade (11–515, Stanley Hand Tools, New Britain, CT). Next, 10,000 MW polyethylene glycol (PEG) (309028, Sigma Aldrich, St. Louis, MO) was placed onto an unused glass coverslip (12–544-E, Fisher Scientific, Waltham, MA), then melted and spread on the coverslip via a wire wrapped around a soldering iron set to 350–400° F (Patel et al., 2015; Supplementary Figure 3B). Mote bases were placed onto the cooled PEG layer with the topside facing up and the surrounding PEG was melted again so that the base would sink and become flat against the coverslip. This positioning ensured that the topside was level during fiber placement. Mote bases were placed with enough spacing between them to prevent disturbing fibers in neighboring motes during placement. Epoxy (Epo-Tek 301, Epoxy Technology, Billerica, MA) was deposited onto the topside via a pulled glass capillary. A 4–6 mm long carbon fiber (T-650/35 3 K, Cytec Thornel, Woodland Park, NJ) was threaded into the base’s hole. Successful placement of the fiber generally required inserting at an angle and bending the fiber slightly (approx. 30°) so that when the fiber straightened after releasing tension, it would be pushed deeper into the hole (Supplementary Figure 4). The epoxy was partially cured overnight at room temperature and then oven-cured at 140°C for 20 min (Huan et al., 2021). In later batches, the bonds between the base and fiber were evaluated for stability by bending or pulling the fibers with forceps. An additional layer of 301 epoxy was added to devices with unsecure fibers and oven-cured shortly afterward (140°C, 20 min). Motes that received additional epoxy and still failed to become sufficiently secure were discarded.

### 2.3 Encapsulating motes with Parylene C

A subset of motes (*N* = 105) was encapsulated in Parylene C (Patel et al., 2015; Lee A. H. et al., 2024). First, motes were released from the securing PEG by melting or dissolution with deionized (DI) water. In parallel, silver epoxy (H20E or H20S, Epoxy Technology) was placed onto a printed circuit board (PCB) (Patel et al., 2016) and shaped to have a dune-like slope (Huan et al., 2021) to hold the motes by their carbon fibers (Supplementary Figure 3C). The epoxy was oven-cured at 140°C for at least 20 min (Patel et al., 2015), after which 660–760 nm of Parylene C was deposited (PDS2035CR) (Patel et al., 2020). Each deposition batch included a glass slide for measuring the Parylene C layer thickness with a stylus profilometer (DektakXT, Bruker Corp., Billerica, MA).

### 2.4 Fire-sharpening carbon fiber tips

Prior to tip sharpening, the fibers were cut to a length of 1050 μm with microsurgical scissors (15003-08 or 15002-08, Fine Science Tools, Foster City, CA) (Patel et al., 2015), where the buffer 50 μm would be burned away during sharpening. During cutting, the coverslip or glass slide holding the devices was placed on its side, held by putty alone or on the side of a wooden block and the cutting length was approximated with an eyepiece reticle in the stereoscope. Generally, motes with fibers of lengths ± 50 μm of the target length were kept.

Tips were then fire-sharpened using a modified procedure reported by Welle et al. (2021) (Supplementary Figure 3D). First, a strip of parafilm (PM996, Bemis Company, Neenah, WI) was cut and placed onto a glass slide (12-550-15, Fisher Scientific) being heated by a PCB warmer (CSI358, Circuit Specialists, Tempe, AZ). Once the parafilm turned clear, a gloved finger smoothened the layer, and motes were then partially stuck into the parafilm with fibers facing up. The slide was placed into a dish filled with DI water and the water level adjusted so that fiber tips were touching the meniscus, which was visualized with a pen camera (TSMS100, Teslong, Shenzhen, China). A butane microtorch with a 1–3 cm long flame was waved along the water such that the hotter central flame briefly pushed down the water meniscus, directly torching the fibers. This was repeated several times before visually inspecting the tip shape through a benchtop microscope. Due to variable carbon fiber tip heights from fiber angles and mote depths in the parafilm, often a subset of fibers sharpened first, and these were removed to prevent oversharpening. The process of torching, inspection, and removal was repeated until all fibers in a batch were sharp.

### 2.5 Assembling insertion devices and mote grids

Insertion devices were assembled using the following steps. First, the head of a nail with a shaft diameter of ∼1.4 mm was cut off and the resulting cross-section was filed or sanded to be orthogonal to the shaft’s length. Then, a glass coverslip, which was cleaved into a square with 1.5–2 mm side length, was epoxied to the nail shaft. Initially Norland Optical Adhesive 61 (NOA 61, Norland Products, Inc., Cranbury, NJ) cured using ultraviolet light was used, but this was replaced by Epo-Tek 301 epoxy heat cured for 20 min at 140°C (Huan et al., 2021).

**FIGURE 2:**
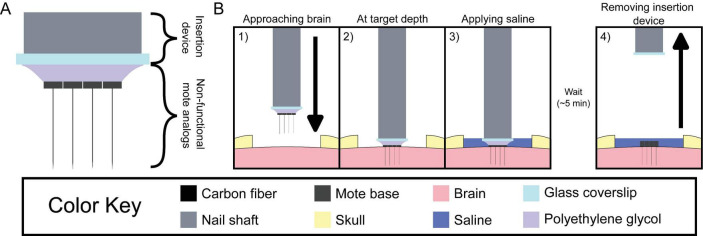
Diagram of carbon fiber mote insertion. **(A)** Profile of a mote grid ready for implantation, with motes held to the device by PEG. **(B)** Procedure for mote implantation: (1) The motes are lowered to the brain. (2) Driving the insertion device downward ceases once the fibers have penetrated the brain and reached the target depth, which is indicated by the bases having reached the brain’s surface. (3) Sterile saline is applied to dissolve the PEG. (4) After waiting for 5–7 min, the insertion device is removed, leaving the motes in place.

Mote grids were composed of motes held onto the glass via PEG (Figure 2A). During mote placement, the insertion nail was held upright with a vial clamp such that the upward-facing glass was parallel to the table. Small flakes of PEG were placed onto the glass and melted by firmly touching the nail under the glass with a soldering iron set to 350–400° F. One or two motes were carried by forceps onto the insertion device near its edge. The iron was then pressed against the nail to melt the PEG while forceps prevented motes from capsizing. If sufficient PEG was present, newly added motes were pulled toward other motes due to surface tension. These steps were repeated until the desired number of motes was present. Periodically, motes were guided in the melted PEG with forceps to facilitate the desired arrangement. Completed grids on the insertion device were stored horizontally. Insertion nails were reused if intact after implantation.

### 2.6 Preliminary mote analog devices

Many of the assembly methods listed above were developed while assembling the first batches of non-functional analogs (*N* = 45 motes) used in proof-of-concept insertions (Table 1 and Supplementary Figure 5). These preliminary devices are relegated to the Supplementary Information, with the exception of reporting their insertion success rate (Table 1), and including their failure modes (Table 2 and Supplementary Table 1). The steps were mostly the same, but none of these preliminary motes were coated in Parylene C nor were the tips fire-sharpened. The fibers were also cut to a target length of 500 μm instead.

**TABLE 1:** Insertion experiment details.

Insertion #	Rat #	Timespan (A/C)	Layout	Reused motes (#)	Coated in Parylene C(Y/N)	Hemisphere	Craniectomy coordinates		Craniectomy shape	Evaluation		Success rate (%)
							M/L	A/P		*S*	*F*	
Proof-of-concept insertion experiments												
1	1	A	3 × 3	0	N	R	1.0–6.0	(-8.0) to (-3.0)	R	8	1	89
2	1	A	3 × 3	0	N	L	0.5–5.0	(-7.5) to (-2.5)	R	4	5	44
3	2	A	3 × 3	0	N	L	0.5–3.5	1.0–5.0	O	9	0	100
4	3	C	3 × 3	0	N	R	0.5–2.5	1.5–5.5	I	9	0	100
5	3	C	3 × 3	0	N	R	0.5–2.5	1.5–5.5	I	9	0	100
Total	–	–	–	–	–	–	–	–	–	39	6	87
Insertion experiments with 1 mm sharpened fibers												
6	4	C	4 × 4	0	Y	L	1.0–4.5	(-7.5) to (-4.5)	T	15	1	94
7	5	C	4 × 4	0	Y	L	1.0–4.5	(-7.5) to (-4.5)	T	16	0	100
8	6	A	5 × 5	0	N	R	0.5–4.0	2.5–6.5	R	22	3	88
9	7	A	5 × 5	0	N	L	0.5–4.0	(-3.5) to (-0.5)	R	25	0	100
10	8	C	4 × 4	0	Y	R	0.5–3.0	1.5–4.0	C	14	2	88
11	9	A	4 × 4	10	N	R	1.5–4.0	(-6.0) to (-3.0)	C	11	5	69
12	10	A	5 × 5	0	N	R	1.0–4.0	2.0–5.0	R	20	5	80
13	11	A	5 × 5	0	N	R	0.5–3.5	1.5–5.5	R	16	9	64
14	12	A	5 × 5	0	Y	L	1.0–4.5	(-5.0) to (-1.0)	R	24	1	96
15	12	A	5 × 5	0	N	R	0.5–4.5	(-5.5) to (-1.5)	R	24	1	96
Total	–	–	–	–	–	–	–	–	–	187	27	87

Mote insertion experiments used various configurations of mote grids in several areas of rat cortex. Relevant experimental details are listed here for more information on how these experiments differed. Proof-of-concept insertions (*N* = 5) with preliminary motes were performed in differing regions of rat cortex with minor differences in technique. Details for proof-of-concept insertions are listed in the first five rows. In the remaining rows, details for insertion experiments with larger grid sizes (4 × 4 and 5 × 5) and with longer (1 mm) sharpened fibers are listed. Craniectomy coordinates are relative to bregma. Timespan column: A, Acute; C, Chronic. Hemisphere column: L, Left; R, Right. Craniectomy coordinates column: M/L, Medial/Lateral; A/P, Anterior/Posterior. Craniectomy Shape column: R, Rectangle; O, Oval; I, Ice cream cone; T, Trapezoid; C, Circle. Evaluation and success rate columns: S, Success; F, Failure.

**TABLE 2:** Failure modes attributed to incomplete mote insertions.

Classification	Surgical complication	Methodology	Brain topography	Mote interaction	Total
Failed entirely – no fiber in brain (# motes)	15	7	4	1	27
Mostly inserted – most but not all fiber in brain (# motes)	1	5	5	6	17
Total	16	12	9	7	44

Motes that failed to insert entirely (*N* = 27 motes) and motes where the fibers mostly inserted but some portion of fiber was outside the brain (*N* = 17) were grouped into four main categories according to the primary failure mode for incompletely inserting. Each count is the number of motes associated with that failure mode (columns) by insertional outcome (rows).

### 2.7 Evaluation of assembled mote grids

**FIGURE 3:**
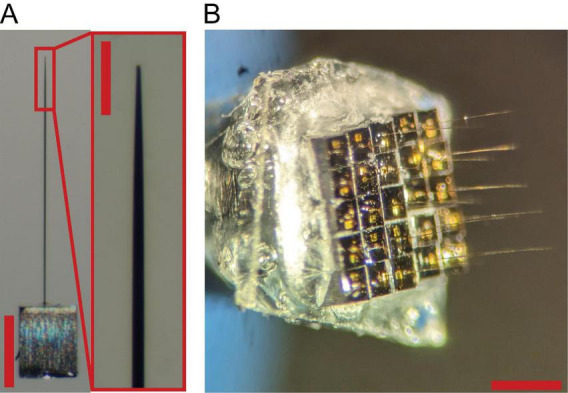
Photos of completed motes. **(A)** Close up view of a completed non-functional analog mote without Parylene C encapsulation. The inset shows a close-up view of the sharpened tip. Scale bar: 300 μm. Inset scale bar: 50 μm. **(B)** A completely assembled 5 × 5 non-functional analog mote grid. The grid is rotated slightly to show some bases (left) and fibers (right) in focus. Scale bar: 500 μm, estimated from mote dimensions.

Once a grid was assembled, it was imaged under a benchtop microscope (SMZ645 or SMZ745, Nikon, Tokyo, Japan) using an eyepiece mounted camera (MU1803-HS, AmScope). For illustrative views only (Figure 3B), some images were captured by a mobile phone camera (Pixel 6, Google, Mountain View, CA) through the eyepiece. To capture the lengths and angles of the carbon fibers (*N* = 3 4 × 4 grids, *N* = 6 5 × 5 grids, *N* = 193 fibers), the grid was oriented with fibers parallel to the plane of focus and a video was captured using the AmScope camera while manually adjusting the focus throughout the device. This simulated volumetric imaging commonly used with an automated stage (e.g., Zuschratter et al., 1998). This process was repeated after rotating the grid around the insertion nail axis by 90°. A calibration slide (0.01 mm/division) (MR095, AmScope) was imaged for scaling. The videos were loaded in ImageJ (Fiji distribution) (Schindelin et al., 2012) and a line was fit from each fiber’s tip to its base for both videos. Lines were also fit to the top edges of mote bases in the closest row and these angles averaged to correct in-plane rotation of the grid. Fibers that were too difficult to distinguish in the videos were excluded. Basic trigonometry was performed via MATLAB (MathWorks, Natick, MA) scripts to determine the fiber lengths and angles in three dimensions, where the angle was defined as the deviation from the vector orthogonal to the mote base’s fiber-side.

Top-down images of completed mote grids were captured similarly for measuring mote spacing. Quadrilaterals were fit to the fiber-side faces of the bases using the polygon selection tool in ImageJ. Quadrilateral centroids were exported to MATLAB, and the distances between a given centroid and the centroids immediately adjacent but not diagonal to it were determined as the pitches. E.g., for a mote centrally located in the grid, four pitches were measured. Across *N* = 6 4 ×4 grids and *N* = 6 5 × 5 grids, 384 pitches were measured. 3.6% of these pitches were shorter than the mote horizontal side length (240 μm), which we attributed to measurement and/or fabrication error.

### 2.8 Implantation of carbon fiber non-functional analog motes

The mote implantation procedure was performed in *N* = 12 male rats weighing 341–628 g; both Sprague-Dawley (*N* = 5) and Long-Evans (*N* = 7) strains were used. The surgical steps for both non-survival (acute) and survival (chronic implant) procedures followed previously published methods (Patel et al., 2016; Letner et al., 2023) until mote implantation steps. For survival surgeries, mote grids were ethylene oxide gas sterilized (*N* = 5) and sterile surgical technique was followed. General anesthesia was provided with isoflurane at 5% (v/v) and 1–3% (v/v) for maintenance. Carprofen (5 mg/kg) was injected subcutaneously as an analgesic. During the procedure, temperature, breath rate, and anesthetic depth were continually monitored. The animal was transferred to a rat stereotaxic frame and the scalp was shaved and sterilized with 1% betadine and then 70% ethanol (v/v) before performing a midline incision. Tissue covering the skull was pulled away with hemostats, and the skull cleaned with 3% hydrogen peroxide followed by saline. A skin marker pen was used to mark intended craniectomy boundaries and, if applicable, bone screw coordinates. In survival surgeries, 6–7 tap holes were drilled for stainless steel (19010-00, Fine Science Tools, *N* = 3 rats) or polyetheretherketone (PEEK) (PKMCK164, High Performance Polymer, Didcot, United Kingdom, *N* = 1 rats) bone screws. Bone screws (1ZY93, Grainger, Lake Forest, IL) were also implanted during *N* = 1 non-survival implant. A craniectomy was then made with a surgical drill and rongeurs. Approximate stereotaxic locations and shapes of each craniectomy are listed in Table 1. To record videos of the insertion from the side, 1–2 pen cameras (TSMS100, Teslong), held by the provided stands, vial clamps, or Flexbars (18032, Flexbar Machine Corporation, Islandia, NY), were placed facing the craniectomy and controlled by laptops. The shaft of the insertion device was then fastened to an electrode holder (M3301EH, World Precision Instruments, Sarasota, FL) held by the manipulator arm of the stereotax frame, and then lowered to the position where at least one carbon fiber touched the dura mater for zeroing the dorsoventral axis. The arm was then retracted upward and durectomy performed with a 23G needle and/or microsurgical scissors. Gel foam (SP40400, Braintree Scientific, Braintree, MA) soaked in sterile saline was placed in the craniectomy to avoid desiccation when not directly interacting with the brain.

**FIGURE 4:**
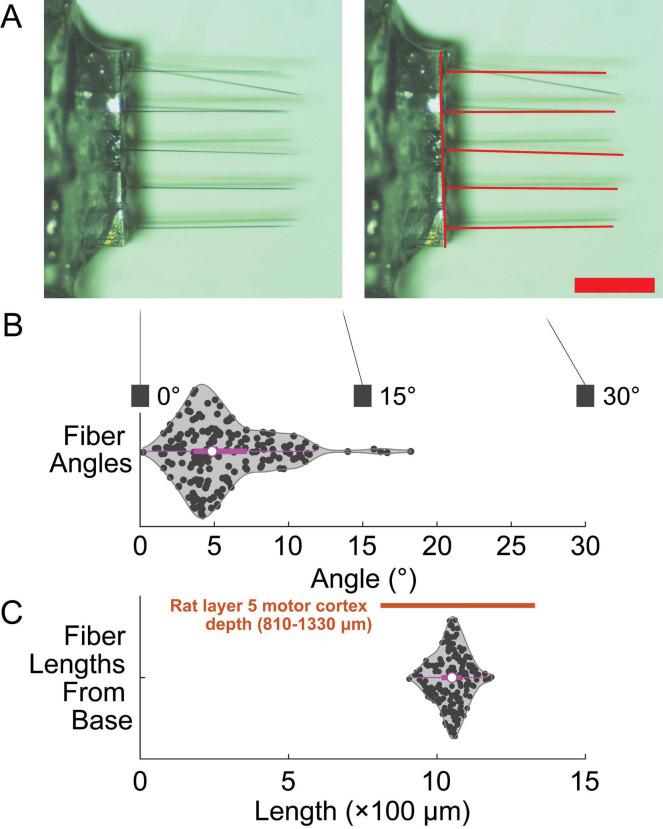
Measuring successful carbon fiber mote fabrication. **(A)** Mote carbon fiber angles were defined as the fiber’s deviation from the vector normal to the face of the mote base from which the fiber extends. These angles and lengths were measured from videos filmed while adjusting the focus plane throughout the grid and fitting lines to the fibers. Example frame used for measurement (left), with annotated image (right) that had lines fit to fibers (horizontal) and mote top faces (vertical). The latter was used to calibrate the overall angle of the grid for a given video. Scale bar: 500 μm. **(B)** Violin plot of *N* = 193 measured fiber angles, 5.7 ± 3.1° (X̄ ± S). **(C)** Violin plot of measured fiber lengths (*N* = 193), 1048 ± 51 μm (X̄ ± S). The range for rat layer V motor cortex (Skoglund et al., 1997), the target region and depth, is shown for comparison.

At this point, the steps specific to mote implantation were performed (Figure 2B). The pen camera(s) started recording and the surgeon’s point of view was recorded through the surgical scope (OPMI CS-NC, Carl Zeiss AG, Oberkochen, Germany) using an attached camera (HVD-37A, Hitachi, Tokyo, Japan: *N* = 5 insertions; MU1803-HS, AmScope: Irvine, CA: *N* = 10 insertions). All movements of the stereotaxic arm, including driving the dorsoventral manipulator axis for insertion, were controlled by manually adjusting the knobs; consequently, the speed of insertion was variable within a given insertion and across all insertions in this study. The stereotaxic arm was quickly lowered to approach the brain (Figure 2B-1). Once fibers touched the brain, the insertion speed was slowed. Sometimes, the motes were retracted a short distance to improve penetration and allow the brain surface to relax. If insertion had started and that there was insufficient room for the insertion device’s glass coverslip or fibers were not penetrating, the probe was repositioned and insertion restarted. The device was driven ventrally to a point where the silicon bases touched the brain and then pushed down some distance further (Figure 2B-2). Sterile saline was then applied via an injection needle to dissolve the PEG (Figure 2B-3) for at least 5–7 min. For proof-of-concept insertions, the dissolution time was typically shorter. In most insertions, the saline was replaced at least once and repeated if necessary. Then, the insertion device was pulled out (Figure 2B-4). In *N* = 2 insertions (insertions 11 and 14), devices that had mostly been inserted were pushed in further using surgical forceps.

### 2.9 Evaluation of mote insertions

Images and videos collected during insertion experiments were carefully reviewed to classify the insertional outcome of each mote that we attempted to insert (proof-of-concept insertions: *N* = 45 motes, larger grid insertions: *N* = 214 motes). These were viewed using a combination of ImageJ, VLC media player (3.0.8) (VideoLAN Org., Paris, France), and Windows (Microsoft, Redmond, WA) media viewing applications, where playback speed was adjusted and segments were viewed repeatedly. Basic image processing techniques were sometimes employed, e.g., gamma correction, to make some video segments clearer. See Supplementary Note 2 for a detailed description of the classification analysis.

Videos were also carefully inspected to attribute probable failure modes that prevented motes from fully inserting via the insertion device. For this qualitative analysis, both proof-of-concept insertions and those with larger grids were considered (*N* = 15 insertions total). See Supplementary Note 3 for details.

### 2.10 Mote spatial arrangement after implantation

Shortly after completing insertion, bird’s eye images were collected using the surgical scope with AmScope camera attached. Only insertion experiments with photos collected after removing the saline were used (*N* = 5 insertions, *N* = 104 motes). Images loaded into ImageJ were scaled by measuring the topside edges of four motes facing the scope and averaging the results. Quadrilaterals were then fit to visible sides. Motes were excluded if they had failed entirely in inserting or had topsides that were partially obstructed by other motes such that fitting a quadrilateral onto them would have been inaccurate. These quadrilaterals were used to determine mote pitch, displacement and tilt after implantation (see Supplementary Note 4).

### 2.11 Statistical analyses

All statistical analyses were performed using MATLAB functions. Most statistics performed were descriptive, such as the mean, standard deviation, and showing distributions as violin plots. The MATLAB function *paramci* calculated 95% confidence intervals of the true mean success rate for insertions. To determine the potential relationship between fiber deviation angle and insertion outcome, multinomial and binary logistic regressions were performed using the *fitmnr* function, which also provided the χ^2^ goodness of fit. When comparing the distributions of post-insertion tilt and displacement measured for motes in this study and Neurograins in Sigurdsson et al. (2020), counts per bin for Neurograins were read from the histograms reported for them, and counts for motes were computed from our measurements using the same binning. Then, both sets of counts were converted to proportions of the total measurements for each design and plotted together.

### 2.12 Figures and graphics

Figures were generated using a combination of software programs. VLC media player (3.0.8) and ImageJ were used for extracting frames from captured videos. MATLAB (versions R2020b and R2024a, with the Violinplot add-on; Bechtold et al., 2021) produced numerical plots. Inkscape (versions 1.1.2 and 1.3) for assembling and producing finalized figures. Videos were edited using a combination of ImageJ, and Kdenlive (version 25.12.0) (KDE e.V., Berlin, Germany).

## 3 Results

### 3.1 Fabrication and assembly of carbon fiber non-functional analogs

This study’s primary goal was to determine whether batches of motes with subcellular-scale penetrating electrodes could be implanted unassisted to cortical depths. To test our proposed implantation method and develop assembly methods for carbon fiber motes, we fabricated carbon fiber non-functional analogs that simulated functional devices with similar dimensions (Figure 1), an approach used in the literature (Sigurdsson et al., 2020). A key consideration was securing the bases during assembly due to their miniscule dimensions (240–300 μm) (Lee A. H. et al., 2020). We found that PEG was suitable when threading carbon fibers into the mote bases (Supplementary Figure 3B), hanging devices by their fibers allowed coating Parylene C on devices in three dimensions (Supplementary Figure 3C), and heated parafilm was suitable for holding non-functional analogs when submerged in water for fire sharpening carbon fibers (Supplementary Figure 3D). We produced at least 230 non-functional analogs with 1 mm carbon fiber length and sharpened tips (example shown in Figure 3A) comprising 11 square mote grids (*N* = 5 4 × 4 grids, *N* = 6 5 × 5 grids) (example shown in Figure 3B). A subset of this total (*N* = 105 motes) were encapsulated in Parylene C. Also, 45 preliminary non-functional analogs with 0.5 mm long carbon fibers were assembled into five 3 × 3 grids to develop the assembly and insertion protocols. From these numbers, we could reasonably conclude that our methods could produce carbon fiber non-functional analog motes in a sufficiently high quantity for insertion testing.

### 3.2 Measuring the quality of non-functional analog mote grid assembly

Next, we sought to measure the quality and consistency of the manual assembly process. Since carbon fiber mote chips will sit on the brain surface and the electrode active site is at the tip (Kozai et al., 2012; Welle et al., 2020), the fiber’s length and angle (Patel et al., 2015) will determine the maximum depth of a mote’s interface. Also, since fibers were manually placed (Supplementary Figure 4), we expected some deviation from perpendicular. Therefore, we measured the deviation angles and lengths of fibers in completed mote grids (*N* = 3 4 × 4 grids, *N* = 6 5 × 5 grids) from videos captured through benchtop microscopes. Figure 4A shows a mote grid with a representative distribution of deviation angles along with line annotation of the closest row of motes. We note that even with a maximum deviation of 10.1°, all 25 motes in this grid inserted into cortex (see Insertion 9 in Table 1). Across all motes, fibers deviated on average only 5.7° ± 3.1° (X̄ ± S) from perpendicular (Figure 4B) and were 1048 ± 51 μm (X̄ ± S) in length (Figure 4C). The tip’s implantation depth is expected to be 7 μm less than this total length on average due to the minimal deviation angles. As Layer 5 (L5) in rat motor cortex spans a depth of 810–1,330 μm (Skoglund et al., 1997), these fibers were capable of comfortably reaching depths suitable for sampling L5 pyramidal neurons once implanted.

**FIGURE 5:**
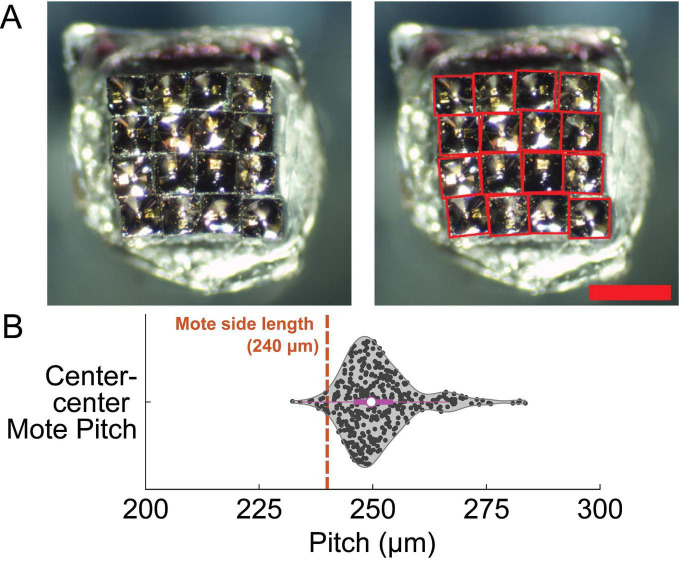
Measuring mote grid spacing after assembly. **(A)** The pitch of a completed grid was measured by fitting quadrilateral ROIs to the motes from top-down images and calculating the distances between centroids. A representative image (left) with fit quadrilaterals shown in red (right). Scale bar: 500 μm. **(B)** Violin plot of *N* = 384 pitches measured from *N* = 12 grids (*N* = 6 4 × 4s, *N* = 6 5 × 5 s), 251 ± 8 μm (X̄ ± S). The mote side length (240 μm) is shown for comparison.

We observed early on that the motes readily aggregated together when partially submerged in the PEG used to affix them to the insertion device. This is shown in Supplementary Video 1 in two ways: first, when a mote base placed onto an insertion device is pushed into heated PEG, it is drawn toward the other bases; and second, the grouped mote bases remain aggregated in heated PEG when pushed. This aggregation minimizes space between motes and enables an electrode density close to the theoretical maximum based on mote dimensions. We therefore prepared all grids in this study with motes aggregated as closely as possible. To determine the resulting pitch, we captured top-down images of mote grids (*N* = 12 grids, *N* = 6 4 × 4 grids and *N* = 6 5 × 5 grids) and measured the center-center pitch from the fiber-sides that were visible (Figure 5A). The resulting pitch (251 ± 8 μm, *N* = 384 distances) was only 11 μm larger on average than the motes’ nominal side lengths (240 μm) (Figure 5B).

### 3.3 Successful insertion of carbon fiber non-functional analog motes

**FIGURE 6:**
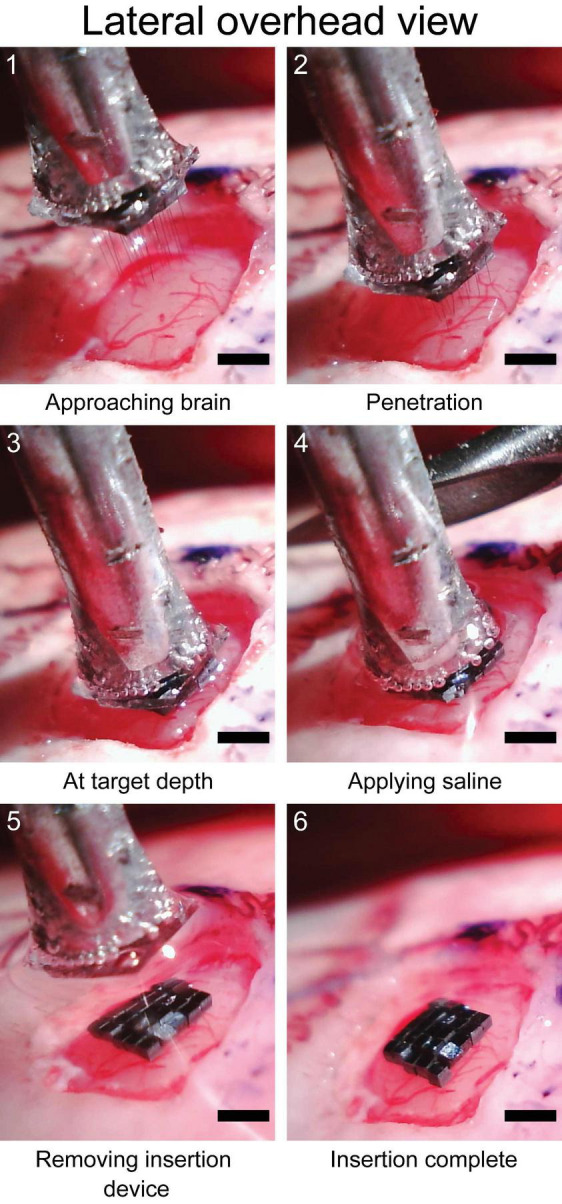
Insertion of carbon fiber non-functional analogs into rat cortex. Frames, ordered alphabetically, extracted from videos captured during an exemplary implantation of a 5 × 5 grid where all 25 motes successfully implanted, captured from a lateral overhead view. See Vid. 2 for source footage. Scale bar: 1 mm.

**FIGURE 7:**
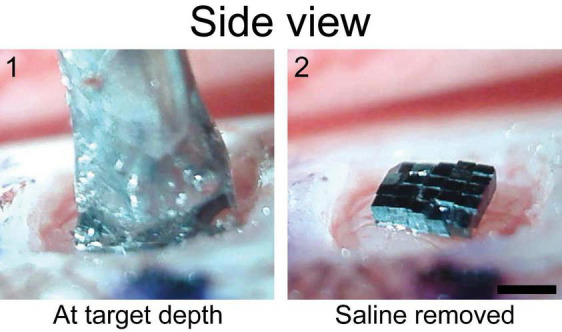
Frames extracted from a side view approximately level with the craniectomy for the insertion shown in Figure 6. See Vid. 3 for source footage. Scale bar: 600 μm.

Having assembled non-functional analog motes, we sought to evaluate how they, and by extension our proposed functional designs, could be implanted into the brain. Using priorities defined by the literature, we aimed to design a procedure with the following attributes: 1) efficient, i.e. inserting tens to hundreds of electrodes simultaneously (Sigurdsson et al., 2020); 2) rapid, in order to avoid unnecessarily increasing neurosurgical duration (Rousche and Normann, 1992; Szostak et al., 2021); 3) facile, in order to avoid further complicating neurosurgery (Rousche and Normann, 1992; Szostak et al., 2021); and 4) minimally invasive to reduce insertional trauma (Obaid et al., 2026). The resulting procedure used PEG to hold devices onto an insertion device (Sigurdsson et al., 2020; Khalifa et al., 2021a; Yamashita et al., 2022) while mote bases were lowered to the surface and the fibers penetrated the brain. Once in place, the devices were released via dissolution of the PEG after exposure to saline over the course of seconds to minutes (Figure 2B). We validated this method *in vivo* by implanting square mote grids (*N* = 15) of multiple configurations into rat cortex (Table 1). To illustrate, we show video frames captured during the insertion of a 5 × 5 mote grid when all 25 devices successfully implanted. Figure 6 and Supplementary Video 2 show the procedure from an overhead point-of-view, whereas Figure 7 and Supplementary Video 3 show the procedure from a viewpoint level with the craniectomy. As these images demonstrate, the carbon fibers penetrated the brain and retained a nearly square configuration once the insertion device and saline were removed.

As each procedure entailed the implantation of multiple motes simultaneously, we defined the success rate for an insertion test as the sum of outcomes observed for each mote attempted. For proof-of-concept insertions using preliminary motes, we implanted grids with low mote counts (*N* = 5 3 × 3 grids) that had short fibers (∼500 μm) and blunt tips. Since 39/45 (87%) preliminary motes implanted successfully (Table 1 and Supplementary Figure 5), these initial successes motivated increasing the grid size and fiber length in subsequent insertions (*N* = 10) to more closely simulate functional device configurations. Overall, larger grids consisting of 16 and 25 motes and 1 mm long fibers with sharpened tips also successfully inserted into the brain. Across 10 insertions (*N* = 4 4 × 4 grids, *N* = 6 5 × 5 grids), 187 out of 214 motes inserted successfully for an overall success rate of 87% (Table 1) and a mean success rate of 87 ± 12% (X̄ ± S). Given these outcomes, we estimate that this procedure has a true success rate of 78–97% with 95% confidence (*N* = 10 insertions). While we report all failures for completeness, these success rates include attempts that failed due to conditions specific to the small craniectomies used in rodents, such as the insertion device colliding with the skull edge of the craniectomy (*N* = 1 insertion) and attempting to insert motes into residual dura (*N* = 3 motes). These conditions would be absent in large mammals or clinical settings where craniotomies are an order of magnitude larger. Excluding these attempts yields an overall success rate of 92% (171/186 motes), and an estimated true success rate of 83–99% (*N* = 9 insertions) with 95% confidence. Bird’s eye views of these insertions along with diagrams of their insertion classifications are shown in Supplementary Figures 6–8. Also, Supplementary Figure 6 illustrates the classification of a successful grid insertion that was representative of most insertions, and Supplementary Figure 7 shows classification of an insertion with poor outcomes.

### 3.4 Characterizing failure modes observed during carbon fiber mote insertion

Although the success rate of implantation was high, we identified several failure modes during these insertions to improve future implants. Using the imaging collected during implantation surgeries (*N* = 15 insertions, 259 motes), we first identified motes that failed entirely to insert (*N* = 27) or mostly inserted with short fiber lengths visible outside of the brain (*N* = 17). We make this distinction because in two experiments, we pushed in motes that had mostly inserted by simply nudging them with forceps, as shown in Vid. 4 and Supplementary Figure 9. Next, we categorized these attempts by failure modes using surgical videos and notes. The full list of putative failure modes and their frequencies is presented in Supplementary Table 1, while counts according to overarching categories for these failure modes are shown in Table 2. Surgical complications, primarily the result of surgeon error, accounted for the highest proportion (36%). Attributes of our assembly and insertion methodology accounted for the second highest proportion (27%), with buckling and early PEG release as common failure modes. These were followed by brain topography (20%), where dimpling from large blood vessels and swelling were the most common. Mote interactions, such as mote collisions, accounted for 16%.

**FIGURE 8:**
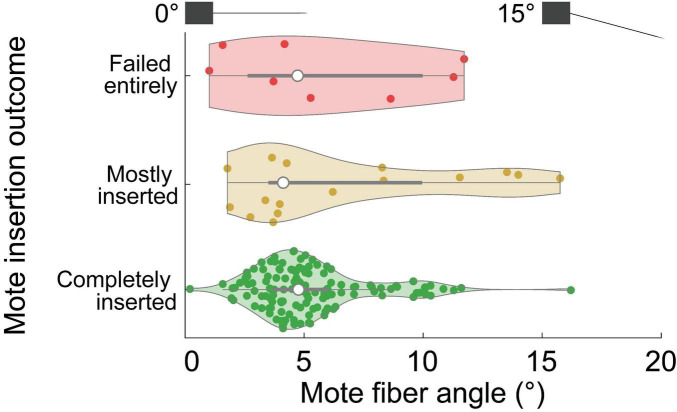
Mote fiber angle categorized by insertion outcome. Carbon fiber angles measured after grid completion and prior to implantation (see Figures 4A,B) for *N* = 7 mote grids were categorized by the insertion outcome for the corresponding mote (*N* = 154 angles). Angles for motes that failed to insert: 5.9 ± 4.2° (X̄ ± S), *N* = 8. Angles for motes that mostly inserted: 6.7 ± 4.6°, *N* = 16. Angles for motes that successfully and completely inserted 5.3 ± 2.5°, *N* = 130. Motes that were excluded from insertion outcome classification are not shown (*N* = 3).

We next investigated whether the carbon fiber deviation angles measured after grid completion (Figures 4A,B), which approximated the fiber angle relative to the cortical surface, could negatively influence insertion success (Massey et al., 2019). Figure 8 shows the deviation angles for motes in *N* = 7 mote grids (*N* = 154 total motes) categorized by insertion success. Interestingly, the angles associated with motes that failed entirely (5.9 ± 4.2°, X̄ ± S *N* = 8 motes) and those that mostly inserted (6.7 ± 4.6°, *N* = 16 motes) were comparable to those that fully inserted (5.3 ± 2.5° X̄ ± S, *N* = 130 motes). Moreover, when we used multinomial logistic regression to determine whether deviation angle might be a factor in insertion outcome, we found that the angle was a nonsignificant predictor (*p* = 0.2, χ^2^ goodness of fit) at these small fiber deviations (<16°). Fiber angle was still nonsignificant even when failed and mostly inserted motes were pooled (*p* = 0.09, χ^2^ goodness of fit). Overall, these results suggest that fiber angle may not be the primary factor in insertion outcome given the deviation angles in this study.

### 3.5 Device arrangement after implant

**FIGURE 9:**
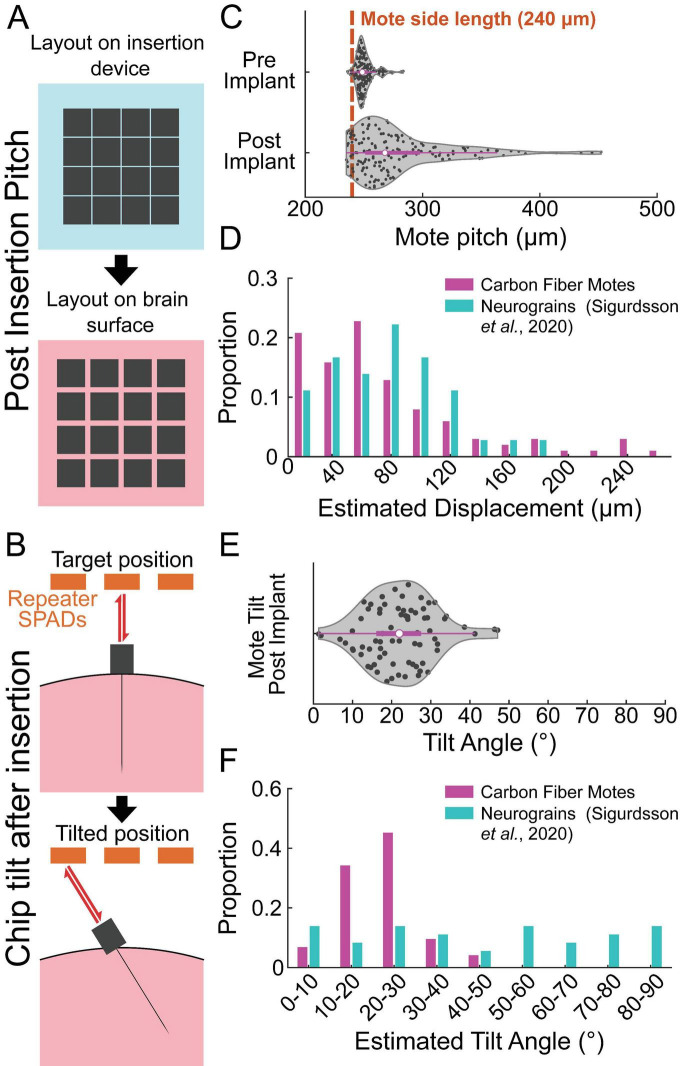
Carbon fiber mote spatial arrangement immediately after implantation. **(A)** Cartoon illustrating an increase in mote pitch after implantation (bottom) relative to the pitch on the insertion device (top). **(B)** Cartoon illustrating how motes tilting on the brain can affect optical coupling (red arrows) with the repeater unit. Top: an ideally implanted mote with no tilt. Bottom: more realistically, after implantation a mote will tilt, which could point to other SPAD detectors (orange boxes). **(C)** Pitch measurements for *N* = 5 mote grids before (251 ± 8 μm, X̄ ± S, *N* = 168 pitches) and after insertion (281 ± 43 μm, *N* = 153 pitches). The mote side length (240 μm) is shown for comparison. **(D)** Comparison of carbon fiber mote displacement after implantation (65 ± 55 μm, X̄ ± S, *N* = 101 motes) to that measured for non-functional Neurograin analogs implanted intracortically into rat cortex in acute conditions (Sigurdsson et al., 2020). **(E)** Tilt angles measured for carbon fiber motes on the brain surface were 22 ± 9° (X̄ ± S, N = 3 insertions, *N* = 73 motes). **(F)** Comparison to tilt angles measured for non-functional Neurograin analogs implanted intracortically into rat cortex in acute conditions (Sigurdsson et al., 2020). **(D,F)** Values for Neurograins were read off of the histograms published by Sigurdsson et al. (2020). Histogram counts were converted to proportions for direct comparison.

Since NIR communication is “line of sight” by definition, where a mote’s topside points may affect its capacity to harvest power and communicate with the repeater unit (Moon et al., 2021; Miziev et al., 2024). Therefore, we calculated motes’ arrangement immediately after implantation by measuring their changes in position (Figure 9A) and tilt from horizontal (Figure 9B), metrics defined previously in the literature (Sigurdsson et al., 2020; Khalifa et al., 2021a). To do so, we collected bird’s eye photos of motes situated on the brain’s surface and manually fit quadrilaterals to the topsides of motes that had fully or mostly inserted (*N* = 3 5 × 5 grid insertions, *N* = 2 4 × 4 insertions, *N* = 104 motes). The resulting pitch was 281 ± 43 μm (X̄ ± S, *N* = 153 pitches), a 12% (31 μm) increase in average pitch from the 251 ± 8 μm (*N* = 168 pitches) measured after grid assembly (Figure 9C). We used a subset (*N* = 3 5 × 5 insertions, *N* = 73 motes) of these quadrilaterals to determine the tilt angles for implanted motes, which averaged 22 ± 9° (X̄ ± S) as shown in Figure 9E. Supplementary Figure 10 shows the annotated source images with corresponding angles for easier visualization of the tilt measurement. To compare these results to prior work, we plot mote displacement (65 ± 55 μm, X̄ ± S, *N* = 101 motes) and tilt against similar measurements reported for Neurograin chips acutely implanted into rat cortex (Sigurdsson et al., 2020) in Figures 9D,F, respectively. These histograms show that mote displacement and tilt measurement distributions skewed lower compared to those measured for Neurograins. Similarly, the percentage of carbon fiber motes that were displaced more than 200 μm was only 5% and the percentage that tilted more than 45° was only 3%, which were lower than the proportions observed for Microbead chips acutely implanted into rat cortex (9 and 36%, respectively) (Khalifa et al., 2021a). Overall, these results suggest that carbon fiber motes may retain their arrangement throughout the implantation procedure to a greater degree than other chips that are implanted inside the brain.

## 4 Discussion

Neural dust offer a solution to the hardware challenges facing the conventional wired intracortical arrays currently used in human BMIs, namely safely accommodating many recording channels (Nurmikko, 2020). However, current dust chips are still much larger than neurons, and therefore placing them entirely intracortically may cause an adverse FBR and displace several neurons (Khalifa et al., 2021b; Lim et al., 2022; Lee J. et al., 2024). Here, we evaluated whether neural dust could be implanted in batches using an approach that is less disruptive to the brain. We assembled and implanted non-functional analog motes that sat on the brain surface and could reach target cortical depths with penetrating carbon fiber electrodes. Our implantation method could implant up to 25 motes into rat cortex quickly and simultaneously with an overall success rate of 87% (187/214 motes) such that only the 6.8 μm carbon fiber occupied brain volume. Moreover, our observation that motes that had successfully inserted also retained their position on the brain surface with respiration and saline removal suggests that this procedure can deliver motes with some degree of security. To illustrate how this approach is advantageous, we can estimate the minimum number of neurons that might be displaced by a mote implanted using either method. A single cubic chip with a 250 μm edge length (1.6 × 10^–2^ mm^3^ volume) implanted to depth could displace approximately 1,100 neurons in mouse motor cortex (7.5 × 10^4^ neurons/mm^3^ on average; Herculano-Houzel et al., 2013). This estimate excludes displacement from the tract made during implantation, which depends on the target depth and insertion aid used. Conversely, the same chip with a 1 mm long carbon fiber electrode (5.5 × 10^–5^ mm^3^ volume) implanted using our procedure might displace only five neurons instead. This estimate is corroborated by our previous histology surrounding carbon fibers, where neurons closest to implanted carbon fiber tips were merely an average of 1 μm further away than hypothetical fibers placed in non-implanted contralateral cortex (Letner et al., 2023).

Despite the potential advantages of such an approach, we found that we needed to introduce a method for implanting batches of motes onto the cortical surface. Whereas previous works have implanted chips inside the brain in batches (Sigurdsson et al., 2020) or singly in rapid succession (Khalifa et al., 2021a), more recent neural dust work has instead opted for epicortical placement one-by-one (Khalifa et al., 2023; Lee A. H. et al., 2024). This shift is likely due to the FBR observed from implanting devices into the brain directly (Sigurdsson et al., 2020; Khalifa et al., 2021c). An important benefit of our method is the ease and lack of complexity involved for the surgeon. We showed that each batch of motes was implanted via simple micromanipulator movements toward the brain, followed by a simple removal of the insertion device after the PEG dissolved. Combined with the sharpened carbon fibers’ ability to easily penetrate the brain surface (Welle et al., 2021; Richie et al., 2024), including the leptomeninges, our insertion method is simple and does not rely on specific insertion speeds or specialized equipment, e.g., the pneumatic inserter required for UEAs (Rousche and Normann, 1992), nor does it require extraneous technical ability of the surgeon. Another advantage is compatibility with conventional micromanipulators, and therefore the possibility of translation to several similar surgical methods used in humans. This includes using a mechanical arm, frame-based stereotaxy, or a neurosurgical robot (Talairach et al., 1962; Tandon et al., 2019). Furthermore, adjunctive neuronavigation is possible, utilizing preoperative magnetic resonance imaging (MRI) to plan one or multiple implant locations (Kaus et al., 1997). For example, 10–20 stereoelectroencephalography electrodes can be placed in one surgery, taking approximately 5 min per electrode with robotic-assisted trajectories guided by preoperative imaging (Tandon et al., 2019). Avoiding contributing complexity to neurosurgery is critical in enabling the translation and clinical adoption of neural dust (Szostak et al., 2021), and the ease of insertion demonstrated here promotes adopting carbon fibers in other neural dust designs.

Although the success rate when inserting up to 25 motes simultaneously was high, achieving the thousands of channels likely required for high-performance BMIs (Lebedev et al., 2011) may require some improvements to the implant method reported here. Although challenges specific to implanting into rodents contributed to insertion failures that would not be applicable, implanting into large animals will bring new challenges. Behind improving the consistency of surgery and assembly, two factors that will have been improved once motes are in the hands of trained clinical neurosurgeons, we found that brain topography was the next most common failure mode for mote insertion. This suggests that brain surface geometry is a principal determinant in insertion success with epicortical dust. Here, we implanted into rat cortex, which lacks the gyral complexity of large mammals (Hofman, 1985; Kawasaki, 2014). When implanting into large mammals with gyrencephalic brains, the number of motes that can be delivered in a single batch will likely be delimited by the size of the target gyrus, as the surrounding sulci fold the brain such that its surface within a given sulcus will be considerable compared to the axis of insertion. Since the sizes of gyri vary considerably (Kennedy et al., 1998), the number of batches and motes per batch will need to be considered and informed by perioperative imaging in clinical settings to provide adequate coverage of the target brain area. While the target gyrus may limit the area suitable for insertion with the procedure introduced in this work, further optimization in chip design may enable increasing the number of motes that can be implanted at once via further minimization of the chip’s size. It has been hypothesized that an electrode pitch of 40 μm could be sufficient for sampling all neurons in the brain (Kleinfeld et al., 2019). Therefore, decreasing the chip size to accommodate this spacing may be ideal in the long term. Since electrodes as thin as 20 μm have successfully inserted into rat brain with an approximately 24 μm thick layer of PEG encapsulation (Gao et al., 2026), we anticipate that this method may be capable of approaching pitches as low as 68 μm, provided that the “bed of nails” effect does not impede insertion as discussed below. At the same time, multiples of 64-channel UEAs with 400 μm pitch (Nordhausen et al., 1996) have restored complex speech in human BMIs (Willett et al., 2023). Given that these arrays typically fit onto gyri of interest, 8 × 8 grids of carbon fiber motes with 400 μm spacing may be a more appropriate goal for effective BMIs for now. A related challenge may be the brain’s pulsation concomitant with respiration, which is negligible in rats but considerably greater in larger animals. Such movement will render the brain a moving target that may place additional mechanical strain onto the carbon fibers. This can be mitigated by embedding the connection point between the chip and fiber with silicone, which can reduce breakage from shearing forces (Welle et al., 2021).

Furthermore, the greater number of penetrating electrodes may increase the “bed of nails” effect, which can become prohibitive in perforating the pia and escalate insertional trauma from the ensuing compression of dimpling (Rennaker et al., 2005; Boergens et al., 2021). Several methods have been proposed to diminish this effect by facilitating pial penetration (Boergens et al., 2021), including removal of the pia (Paralikar et al., 2006), thinning the pia by laser ablation (Boergens et al., 2021), stiffening the pia (Kralik et al., 2001), sharpening electrode tips (McNamara et al., 2024; Obaid et al., 2026), increasing insertion speed (Rousche and Normann, 1992) or simply increasing electrode spacing (Schwarz et al., 2014). More basic research is required to determine which techniques can most effectively reduce dimpling in general, however, as prior work showing tradeoffs in reducing bleeding and cell disruption can render the best course of action inconclusive (McNamara et al., 2024). Prior simulations suggest that insertion dynamics can become increasingly unpredictable as electrode diameters decrease (Chen et al., 2021), which likely exacerbates inconsistencies seen empirically but also makes predictive modeling challenging.

In this work, we applied one of the aforementioned techniques for reducing the “bed of nails” effect by flame-sharpening the carbon fiber tips, but we still saw dimpling across the whole grid in most cases. This dimpling may have stemmed from a combination of factors, including inconsistent sharpening (Richie et al., 2024) and high electrode density (Schwarz et al., 2014). Qualitatively, we observed that fiber tips that had become closer together on the brain surface than the designed pitch (240 μm), due to fiber bending or non-perpendicular fiber placement in the mote, exacerbated dimpling in that portion of the grid. From these observations, we can infer that a combination of improvements will likely ameliorate the dimpling seen here. For the fibers themselves, increasing their diameters by small amounts (1–2 μm) could decrease fibers bending toward one another considerably due to the fourth-power relationship between diameter and bending stiffness (Stiller et al., 2018). Moreover, increasing the stiffness through thickening the electrode may also enable sharpened carbon fibers to insert deeper than the current reliable insertion limit of 1.5 mm (Richie et al., 2024). Improvements in mote assembly, as discussed below, could increase fibers’ penetrative capacity and reduce error in tip spacing through more consistent tip shaping and fiber angles, respectively. Additionally, fiber density could be reduced by increasing the mote side lengths or by introducing spacers to the insertion device. Furthermore, spreading out the delivery of the motes across time may be a viable solution for mitigating both the bed of nails effect and accommodating the uneven surfaces of gyrencephalic brains by implanting several small batches sequentially, an approach that has been used with NET probes (Zhao Z. et al., 2023) and is compatible with motes’ modular architecture. This is a viable strategy in humans if performed sufficiently quickly, which may be achievable by leveraging the adjunctive SEEG implantation procedure mentioned above (Tandon et al., 2019). While sequentially implanting larger batches, such as 8 × 8 grids, than those used here would be preferable due to its higher efficiency, we anticipate that the batch sizes reported in this work would succeed in large animal models.

A limitation of our approach was that motes were assembled entirely by hand after the ‘chips’ were batch fabricated in the cleanroom. These manual techniques commonly used to fabricate carbon fiber electrodes have previously been considered a barrier to clinical viability due to increased variability in electrode quality and reduced throughput compared to fully automated procedures (Hejazi et al., 2021; Lucio Boschen et al., 2021). Although this variability did not preclude the fibers from having sufficient reach to extend to the target cortical layer, larger fiber deviations did appear to be detrimental to insertion success in some cases, even though these angles were sufficiently small to be a nonsignificant factor overall. To reduce the variability in carbon fiber angles, and to improve their consistency generally, a more repeatable process akin to the automated carbon fiber assembly systems reported previously (Massey et al., 2016; Dong et al., 2023), or at least threading the fiber with a micromanipulator (Khalifa et al., 2023), could be employed. For example, fibers placed robotically into micromachined shanks had deviation angles that were an order of magnitude smaller than those presented here (Dong et al., 2023). Laser cutting and sharpening the tips (Dong et al., 2021) could also improve the consistency in fiber length and electrode shape. Manual assembly also rendered the process more tedious and time consuming, where transferring motes between the substrates that secured them expended the most time. While holding devices in PEG during assembly offered a high degree of freedom, its irregularity hinders implementing a mechanized assembly process and its use precludes gentler handling methods, such as vacuum pickup (Khalifa et al., 2023). Gentler handling in particular will likely be required when assembling functional devices in the future, as the intended geometry achieved by bonding two thinned chips has considerably more fragility than the micromachined silicon prisms simulating the chips in this study. Using a regularly-spaced mold or substrate formed around the mote chips instead of PEG could improve the regularity and the efficiency of the process, and could reduce rough handling, as has been shown when cleanroom processing Neurograins in polydimethylsiloxane molds (Lee A. H. et al., 2020). In any case, we built upon our existing benchtop assembly methods that have produced successful carbon fiber probes in prior work (Richie et al., 2021) as a baseline to motivate the assembly of carbon fiber motes and to provide a baseline for future work.

In addition to mechanization, another critical step toward producing clinically viable carbon fiber motes will be integrating new steps to the assembly process that will enable producing functional motes, which will bring several challenges. For example, this will require the introduction and validation of conductive materials to the junction between the fiber and chip, such as conductive epoxy, and applying a conductive coating to the tip, such as PtIr (della Valle et al., 2021) and poly(3,4-ethylene dioxythiophene):sodium p-toluenesulfonate (PEDOT:pTS) (Patel et al., 2016) as we have used previously. As we electrodeposited these coatings to the tips of wired carbon fiber arrays in prior work, continuing to use these coatings will require either a temporary electrical connection or processing the electrode tips prior to inserting them into the chip. Furthermore, a layer encapsulating the chip that is more robust than Parylene C alone will be required to protect the motes within the body to ensure proper function over decades (Shen and Maharbiz, 2021), which will require additional process steps. While considerable additions to the assembly process and functional validation remain before carbon fiber motes could become a viable electrode array for clinical BMIs, establishing their mechanical and surgical viability here motivates further work into developing them.

## Data Availability

The raw data supporting the conclusions of this article will be made available by the authors, without undue reservation.
