# Supplementary material for: A method for efficient, rapid, and minimally invasive implantation of individual non-functional motes with penetrating subcellular-diameter carbon fiber electrodes into rat cortex
Source: Front Neurosci. 2026 Jul 28;20:1848862. doi: 10.3389/fnins.2026.1848862 (PMC13457210; doi:10.3389/fnins.2026.1848862)
Supplement: Supplementary file 1 [file Data_Sheet_1.docx]

Supplementary Material

# 1 Supplementary Figures

**Supplementary Figure 1.** Cross-sectional diagram of non-functional mote analogs. Non-functional motes were fabricated with conductive holes. For this work, only insertion testing was performed, so the conductive capabilities were not used. Also, many devices were not encapsulated in Parylene C for insertion testing (see Table 1).

**Supplementary Figure 2.** **Assembly steps flow for non-functional carbon fiber mote analogs.** This diagram shows the flow of steps used for assembling non-functional carbon fiber mote analogs after the silicon bases were micromachined in a clean room. All steps are benchtop processes, with the exception of Parylene deposition. See the methods text for more details. Four different types of motes were used in this work: 1) preliminary motes with target lengths of 500 µm and blunt tips, used for proof-of-concept insertions (N=5 insertions), 2) motes with target lengths of 1 mm and sharp tips (N= 5), 3) motes with target lengths of 1 mm and sharp tips, where all but the tip was coated in Parylene C (N=4), and 4) motes with target lengths of 1 mm and sharp tips reused from previous insertions (N=1). The steps above cover the steps for the first three cases (reused motes were simply cleaned), which is why there are multiple pathways to a completed grid of motes. Light red colored arrows and text denote steps that could differ depending upon the type of mote being assembled. PCB = printed circuit board.

**Supplementary Figure 3.** Snapshots from carbon fiber mote assembly. (A) Source non-functional mote analogs produced from cleanroom fabrication. Mote bases were attached to a thick layer of Parylene C (white) on their bottom side deposited prior to singulation. This Parylene C layer in turn was held by a carrier wafer, which was made visible after removing a subset of the mote bases (red arrow). Inset is for closer inspection of nine mote bases. Scale bar: 1 mm; scale bar (inset): 250 µm. (B) During fiber placement, mote bases were held in place on a glass coverslip via PEG. Left shows a top-down view of four motes held in PEG, indicated by red arrows. Insets (right) show a closer view of a mote. To more easily illustrate the PEG (clear and white colors) for this manuscript, black electrical tape is used in the background (left, top right). During assembly, blue tape was used instead (bottom right). Arrows in the insets point to the carbon fiber holes. Scale bar (left): 1 mm; scale bars (right): 200 µm. (C) Motes were suspended from a circuit board by their fibers via silver epoxy to ensure that Parylene C could be deposited on all surfaces. Image captured after Parylene C deposition. Scale bar: 2 mm. (D) Fire-sharpening non-functional motes diagram (bottom) and view from pen camera (top). Motes are submerged in water to protect the fibers except at the tip when exposed to flame from a butane torch. Parafilm melted onto a glass slide is used to hold the motes while submerged since it is not water soluble, unlike PEG. The pen camera is used to monitor the water level via mote reflection so that the meniscus is touching the fiber tips during sharpening. Scale bar: 1 mm. Diagram and procedure adapted from (Welle et al., 2021).

**Supplementary Figure 4.** Threading carbon fibers into mote base holes. Shown here is a three-part action sequence illustrating the motions generally used to thread carbon fibers into the holes of carbon fiber mote bases. First, the fiber is brought to the hole with forceps with a lateral orientation. Once the end of the fiber is at the top of the hole, the fiber is pushed downward and laterally to force the fiber to bend. Once enough tension is applied to the fiber, it springs downward into the hole and the fiber is release from the forceps. Green curved structure: fine tipped forceps. Black line: carbon fiber. Dark grey rectangle: mote fiber base. Light rectangle: hole in mote base.

**Supplementary Figure 5.** Outcomes for proof-of-concept mote insertions. Photos captured after implantation of preliminary mote grids in proof-of-concept insertion experiments. These motes differed slightly from subsequent devices and experiments: these motes were arranged in a 3x3 grid pattern, target fiber length was 500 µm, and the tips were blunt. Additionally, the insertion device was removed in a horizontal motion parallel to the brain surface for insertions 1, 2 and 4, and a mix of horizontal and vertical removal for insertion 3. Out of 45 motes in this dataset, 39 implanted successfully, a success rate of 87%. For each insertion, photos or frames collected post insertion are shown on the left, with a diagram showing insertion success classification on the right.

**Supplementary Figure 6.** Illustrative example of classifying an insertion with representative outcomes. Multiple viewpoints of an insertion shortly after removal of the insertion device: (A) bird’s eye view, scale bar: 750 µm; (B) side view from the anterior edge of the craniectomy, scale bar: 300 µm; (C) side view from the medial (right) side of the craniectomy, scale bar: 400 µm; (D) lateral & posterior overhead (vertically from the bottom left corner of the craniectomy) view, scale bar: 400 µm. Motes that successfully inserted with an estimated 200 µm or less of fiber remaining (N=6) are labelled with arrows and the estimated length of fiber that is still outside of the brain, rounded up to the nearest 100 µm. The mote that entirely failed to insert (N=1) is labelled with an arrow and the letter “X”. (E) Insertion outcome classification diagram for this insertion. The layout matches that shown in the bird’s eye view. Image processing: (A) none; (B) 10 frames of video captured were averaged, then gamma corrected and contrast adjusted in ImageJ (Schindelin et al., 2012); (C) none; (D) gamma corrected in ImageJ.

**Supplementary Figure 7.** Illustrative example of an insertion with poor insertional outcomes. Bird’s eye view (A) and left side view (B) of an insertion with poor insertional outcomes shortly after removal of the insertion device. Motes that completely failed to insert (N=3) are labelled with arrows and the letter “X” and are unambiguously identifiable because these motes fell to their side. Motes that mostly inserted are labelled with arrows and the estimated length of fiber still outside of the brain (N=4), rounded up to the nearest 100 µm. Two of these motes had more than 200 µm of fiber remaining to be pushed in (N=2) and were therefore classified as failures. (C) Diagram showing insertion success classification for this insertion experiment. The layout corresponds to the bird’s eye view. (a) Scale bar: 750 µm. (b) Scale bar: 500 µm. Image processing: (A) none. (B) 10 frames of video captured were averaged, then gamma corrected and contrast adjusted in ImageJ (Schindelin et al., 2012).

**Supplementary Figure 8.** Outcomes for mote grid insertion experiments. Photos captured after implantation of 4x4 (N=4) and 5x5 (N=6) mote grids during insertion testing experiments. To reduce redundancy, the outcomes for insertions 14 and 12 are not shown here because they are included in Supplementary Figures 6 and 7, respectively. For each insertion, a bird’s eye photo is shown after removal of the insertion device (left) and insertion success classification is shown diagrammatically on the right with layouts matching the bird’s eye photos. Scale bars: 750 µm. Note: No image processing was applied to these photos, with the exception of the photo for Insertion 15, which was gamma corrected in ImageJ (Schindelin et al., 2012).

**Supplementary Figure 9.** Example correction of poorly inserted motes. Bird’s eye photos captured of an insertion shortly after the removal of the insertion device (left) and after correction (right). Motes that had completely failed to insert (N=3) were removed via forceps and are labelled “R” (removed) (left). Motes that had mostly inserted with more than 200 µm of non-inserted fiber remaining that were pushed in during correction (N=2) are labelled “F” (fixed) (left). The remaining devices were straightened and, if 0-200 µm of non-inserted fiber remained, pushed in further as part of the procedure. This is from insertion 11, shown in Supplementary Figure 8. Insertion success classification yielded 11 motes that had successfully inserted (69%), and 5 that failed to insert (19%). Correcting the devices that had mostly inserted increased the proportion of fully inserted motes to 81%. Scale bars: 750 µm.

**Supplementary Figure 10.** Visualization of mote tilt angle analysis. The tilt from horizontal for motes that were implanted onto the surface of the brain was calculated by fitting a quadrilateral to the topside of each mote, using Perspective-n-point calculation to determine the mote pose relative to the camera, and then compared to a reference angle defined by the average pose of all motes implanted in that experiment (N=3 insertions, N=73 motes). On the left, source images are shown with the quadrilaterals (yellow) used for the calculation. On the right, grids with the calculated angles are shown. The layout of motes in the angle grids are the same as in the source images (i.e. the top left mote in the source photo is the same as the top left in the grid, etc.). Scale bars: 400 µm. Image for Insertion 15 was gamma corrected in ImageJ.

# 2 Supplementary Tables

| **Failure Mode** | **# Associations** | **Primary Category** |
| --- | --- | --- |
| Buckling | 10 | Methodology |
| Dimpling - blood vessel & swelling | 7 | Brain topography |
| Surgeon error - fast | 6 | Surgical complication |
| Early PEG release on much of array | 5 | Methodology |
| Pia hardened by blood | 5 | Surgical complication |
| Brain pushed down by another mote | 4 | Mote interactions |
| Surgeon error – collision with skull | 4 | Surgical complication |
| Brain blocked by dura | 3 | Surgical complication |
| Evidence of large fiber angle | 3 | Methodology |
| Large fiber angle | 3 | Methodology |
| Mote collision | 3 | Mote interactions |
| Prerelease | 3 | Methodology |
| At edge of swelling | 2 | Brain topography |
| Blood in craniectomy | 2 | Surgical complication |
| Evidence of mote collision | 2 | Mote interactions |
| Horizontal removal of insertion device | 2 | Methodology |
| Not released | 2 | Methodology |
| Blood vessel | 1 | Brain topography |
| Dimpling | 1 | Brain topography |
| Dimpling - blood vessel | 1 | Brain topography |
| Distance to brain | 1 | Surgical complication |
| Evidence of buckling | 1 | Methodology |
| Evidence of loose fiber | 1 | Methodology |
| Evidence of prerelease | 1 | Methodology |
| Evidence of pull out by blood | 1 | Surgical complication |
| Large blood vessel | 1 | Brain topography |
| Loose fiber | 1 | Methodology |

**Supplementary Table 1.** Failure modes for incomplete mote insertions. Failure modes are listed in descending order from the most associations to the least. Since multiple failure modes were attributed to most motes (25/44), the total number of associations in this table is greater than the number of motes assessed. The third column lists the primary category that was associated with each failure mode.

## 2.1 Supplementary Note 1 – Cleanroom Microfabrication of Mote Bases

The source substrate for silicon bases was a 4” p-doped silicon wafer that was 300 µm thick and polished on both sides (2345, UniversityWafer, South Boston, MA). The wafer was coated in a layer of Parylene C approximately 5 µm thick (PDS2035CR, Specialty Coatings Systems, Indianapolis, IN) with AP-174 adhesion promoter. This layer was applied to the wafer backside to later hold the individuated mote analogs. The layer of Parylene C on the wafer’s topside was removed via oxygen plasma ashing (YES-CV200RFS(E), Yield Engineering Systems, Fremont, CA). SPR 220 (3.0) Photoresist (MEGAPOSIT SPR 220 (3.0), Series i-Line; Dow Chemical Co., Midland, MI) applied to the topside followed by deep reactive ion etching (DRIE) (STS Pegasus 4, SPTS Technologies Inc., Newport, United Kingdom) produced a 20, 25 or 30 µm hole in each device. These three hole sizes intentionally differed for determining viable carbon fiber hole sizes. Due to inconsistencies in the DRIE, the hole did not always go through the whole device. A 30 nm passivation layer of Al_2_O_3_ followed by a 10 nm conductive layer of TiN were added to the topside and the hole sidewalls via atomic layer deposition (Fiji ALD, Veeco Instruments Inc., Plainview, NY) to render the hole and topside electrically connected. A 50 x 50 µm contact pad (10 nm Ti, 200 nm Au) was sputtered (Lab 18, Kurt J. Lesker Co., Pittsburgh, PA) onto the topside beside the hole and patterned via the liftoff process, patterned with LOR 10B (Kayaku Advanced Materials Inc., Westborough, MA) followed by SPR 220 (3.0). Singulating the sample into individual mote bases, similar to wafer dicing, was then achieved in three steps: 1) patterning with SPR 220 (3.0) photoresist, 2) portions of the TiN and Al_2_O_3_ layers that covered intended etch lanes for the silicon bases were etched via ion milling (Nanoquest II, IntIvac Thin Film Corp., Fort Collins, CO) 3) the sample was mounted to a 4” carrier wafer with Santovac 5 (Santolubes, LLC; Spartanburg, SC) and singulated via DRIE (STS Pegasus 4). Devices were held in place on the layer of Parylene C for storage indefinitely until removed for assembling a non-functional carbon fiber mote analog (Supplementary Figure 3A).

## 2.2 Supplementary Note 2 – Detailed Mote Insertion Classification

Generally, a small number of motes that failed to insert were easily identified during the procedure as they either sprung away from the insertion site or fell onto their side, which is why one was immediately removed in insertion 6. For insertions with 4x4 and 5x5 grids, some motes were mostly inserted but some portion of the fiber was visibly outside of the brain. Because some of these motes were nearly entirely in the brain (e.g., motes in Supplementary Figure 6) and some were pushed in afterward with forceps (Supplementary Figure 9, Video 4), we made the *post hoc* decision to classify these devices as successes or failures using a threshold on the remaining fiber length. For motes where some portion of the fiber was visible (N=17), we estimated the length of visible fiber in ImageJ, where the image was scaled using nearby motes and the horizontal and vertical scales sometimes differed. This length was rounded up to the nearest 100 µm to compensate for collecting images that were not perpendicular to the motes. If the length was ≤200 µm, the mote was classified as having been successfully inserted. Otherwise, the mote was classified as having failed to insert. We chose this threshold because the fibers had a target length of 1000 µm, and the depth where Layer V begins in rat motor cortex is 810 µm (Skoglund et al., 1997).

There were some cases where a small segment of the fiber was visible in the video but the mote base appeared to otherwise sit on the brain surface. These were classified as successes as this evidence pointed to those fiber segments being visible due to the transparency of the pia. Additionally, motes that were situated in inner rows and columns of the grid may have had some portion of fiber outside the brain, but side views of these devices were blocked by motes in nearby rows or columns. We classified these motes as having successful insertions as the height of their bases was equal to or less than those of motes in outer rows that had been successfully inserted. Overall, N=214 1 mm motes and N=45 preliminary motes were attempted and classified. For proof-of-concept insertions (N=5 3x3 grids), insertion success was evaluated more stringently. Some devices that tilted with qualitatively large angles were classified as failures due to their shorter fiber length.

We note that in the *Successful Insertion of Non-functional Carbon Fiber Mote Analogs* subsection of the results, we report a second success rate that excludes motes where the small craniectomy size used for rodent insertions were related to surgeon error. In one insertion (Insertion 8), N=3 of these motes were excluded due to unsuccessfully removed dura blocking these devices from attempting to insert into the brain. An additional 5x5 mote grid insertion was excluded entirely because the insertion device bumped into the edge of the craniectomy when being driven toward the brain. The force of the collision with the skull was sufficiently high to shear several of the fibers such that they broke off the mote base, which had not been seen before, and was indicative that the insertion was abnormal. These motes were included in our analysis characterizing failure modes of insertion (Table 2, Supplementary Table 1), but were excluded from our assessment of how fiber angle contributed to insertion outcome (Figure 8).

## 2.3 Supplementary Note 3 – Failure Mode Analysis

First, motes were categorized into whether they had mostly inserted (N=17) with some portion of the fiber visible or had entirely failed to insert (N=27). It is important to note that motes that had mostly inserted with spans of fiber that were ≤200 µm in length and were classified as successful above were included in this analysis as motes that had mostly inserted. Devices were carefully tracked in insertion videos to determine failure modes that factored into their insertion outcome. For the majority of these motes (25/44 motes), multiple failure modes could be attributed to a given mote’s outcome, e.g., a device that started to release early but also was pushed by another mote, so all possible failure modes were attributed to each device. These failure modes were then ranked in order of estimated effect on the insertion outcome for that mote. This ranking varied in subjectivity, as some rankings could be clearly deduced from the videos while for others what could be seen in videos was more ambiguous. This ranking is why the frequencies of all identified failure modes, shown in Supplementary Table 2, add up to a number greater than the total number of motes assessed (N=44). Each failure mode was then assigned to one of four primary categories: methodology, brain topography, mote interactions, or surgical complication. Each mote assessed was then assigned one of those four primary categories according to the primary category of the highest ranked failure mode, which is shown in Table 2.

## 2.4 Supplementary Note 4 – Detailed Methods for Measuring Mote Arrangement

Continued from Section *2.10 Mote Spatial Arrangement After Implantation*. To measure mote pitch after insertion (Figure 9C), quadrilaterals manually it onto the topside faces of CF mote bases implanted onto the brain were used. The Euclidean distance between the centroid of each quadrilateral and neighboring quadrilaterals was determined using the same process as that used for pre-insertion pitch measurement and used as the post-insertion pitch.

To estimate mote displacement after insertion, pictures and insertion videos were used to determine the change in grid orientation between top-down photos captured after grid completion and the photo captured after insertion. Centroids of quadrilaterals fit for pre-insertion pitch measurements were rotated and flipped vertically to match their post-insertion arrangement. Both pre-insertion and post-insertion centroids were then centered by either the centroid of the central mote (5x5 grids) or a corner point of a centrally located device (4x4 grids). Both sets of coordinates were rotated, where the angle was defined by the slope between two mote centroids that were representative of the overall rotation of the grid post insertion. With the grids aligned, the displacement was measured as the Euclidean distance between each mote’s pre-insertion centroid position and its post insertion centroid position. Since the displacement for the center mote in 5x5 grids was 0 µm, these center motes were excluded from the final distribution (N=3 motes).

To determine mote tilt, the vertices of each quadrilateral fit to the top face were imported into MATLAB using a custom script based upon ImageJ’s region of interest code (accessible on GitHub at https://github.com/imagej/ImageJ/blob/master/ij/io/RoiDecoder.java). They were rotated according to the rotation measured during displacement estimation and translated such that the bottom left corner of the grid was at the origin. Perspective-n-point estimation (Lu, 2018) was performed via the *solvePnP* function of the OpenCV library (Bradski, 2000) for each mote to determine its pose relative to the camera, where perfect top-down coordinates were used as the world coordinates. The rotation components of the poses of all motes in the insertion were averaged (Bruno Luong. RotationMean [https://www.mathworks.com/matlabcentral/fileexchange/134037-rotationmean], MATLAB Central File Exchange) for use as a reference frame, as the pose of each mote was offset since the surgical scope was not directly parallel to the surface of the brain. The tilt for each mote was calculated by determining the rotation required to rotate a mote with the reference orientation to the pose estimated by *solvePnP* and then extracting the rotation relative to the positive z-axis in the reference frame using the MATLAB *tilt* function. One insertion was excluded because the surgical camera was positioned at too large of an angle for the rotation averaging to produce a viable reference given the assumptions made about the world coordinates. Another insertion was excluded because the resultant angles were clearly incorrect, which we attribute to the poorer quality of the image. In total, N=3 insertions were used for mote tilt determination (N=73 motes).

## 2.5 Supplementary Note 5 – Caveats in Methods

### 2.5.1 Relevant to assembling Mote Grids

In N=1 4x4 mote grid, N=10 motes were reused after a previous implantation (these were from insertion 8, reused in insertion 11). Prior to placing these motes in a mote grid, they were soaked overnight in an enzymatic detergent solution (approximately 1.5% in deionized water, vol/vol) (Enzol, 2252, Advanced Sterilization Products, Irvine, CA). Also, a 4x4 grid that had been sterilized with ethylene oxide gas was upgraded to a 5x5 with new motes (insertion 14). For N=2 mote grids, additional PEG was added at the mote bases to increase stability during implantation after observing that premature PEG release was a failure mode.

### 2.5.2 Regarding preliminary mote analog devices

Some of these devices were also held in place using 2050 MW PEG (295906, Sigma Aldrich) instead, a higher temperature setting on the soldering iron was used, and the fibers were dipped in DI water prior to insertion to remove excess PEG. In one batch, 301 epoxy and 353ND-T (Epoxy Technologies) were incorrectly mixed together, but fibers were sufficiently fastened for insertion testing.

### 2.5.3 Regarding insertion

In N=1 rat, a second grid insertion was performed in the same craniectomy (insertions 4 & 5) to replace the previous grid, which had been mistakenly ruined by excess cyanoacrylate glue (Vetbond, cat. # B00016067, 3M, Maplewood, MN) as part of a chronic experiment. For N=2 rats, 2 insertions were performed in separate craniotomies, accounting for N=4 insertions. For N=1 experiment, the insertion was performed after a spinal cord implantation performed for another study. This rat had initially been anesthetized with a cocktail of ketamine (90 mg/kg) and xylazine (10 mg/kg) for induction followed by maintenance doses of ketamine (30 mg/kg) every 20-30 minutes. Once the spinal cord component was completed, the mote insertion continued as described above.

# 3 Video Legends

**Video 1.** Motes readily aggregating in PEG. Video showing how motes will aggregate in molten PEG. A mote base is placed onto the glass of an insertion device. The soldering iron is then held to the nail shaft and the PEG is melted. Once the mote base is nudged into the molten PEG, it is pulled toward the other mote bases in PEG due to surface tension. The group of motes is prodded with forceps to show how they still stick together when PEG is molten. Bases without fibers were used for demonstration purposes. Video is shown at regular speed. Scale bar: 500 µm.

**Video 2.** Mote implantation (vertical view). Video showing an exemplary mote implantation where all 25 motes in a 5x5 grid successfully implanted into rat cortex. This video was captured from a pen camera looking downward at the craniectomy. The video is sped up by a factor of 8, and much of the saline application step is removed to reduce runtime. Frames from this video are displayed in Figure 6. Scale bar: 750 µm.

**Video 3.** Mote implantation (side view). Side view of the same implantation shown in Video 2. The video is sped up by a factor of 8, and much of the saline application step is removed to reduce runtime. This video lines up in time with Video 2. Frames from this video are displayed in Figure 7. Scale bar: 750 µm.

**Video 4.** Pushing in Motes That Had Mostly Inserted. Side view of pushing motes in further with forceps to complete insertion for a 5x5 grid. The device with the large tilt did not push in all the way, but was pushed in deeper. Video is shown at regular speed. Scale bar: 600 µm.

# 4 Supplemental References

Bradski, G. (2000). The OpenCV Library. *Dr. Dobb’s Journal of Software Tools*. Available at: https://opencv.org/

Lu, X. X. (2018). A Review of Solutions for Perspective-n-Point Problem in Camera Pose Estimation. *J. Phys.: Conf. Ser.* 1087, 052009. doi: 10.1088/1742-6596/1087/5/052009

Schindelin, J., Arganda-Carreras, I., Frise, E., Kaynig, V., Longair, M., Pietzsch, T., et al. (2012). Fiji: an open-source platform for biological-image analysis. *Nat Methods* 9, 676–682. doi: 10.1038/nmeth.2019

Skoglund, T. S., Pascher, R., and Berthold, C. H. (1997). The existence of a layer IV in the rat motor cortex. *Cerebral Cortex* 7, 178–180. doi: 10.1093/cercor/7.2.178

Welle, E. J., Woods, J. E., Jiman, A. A., Richie, J. M., Bottorff, E. C., Ouyang, Z., et al. (2021). Sharpened and Mechanically Durable Carbon Fiber Electrode Arrays for Neural Recording. *IEEE Trans. Neural Syst. Rehabil. Eng.* 29, 993–1003. doi: 10.1109/TNSRE.2021.3082056
